# The potential value of serum pepsinogen for the diagnosis of atrophic gastritis among the health check-up populations in China: a diagnostic clinical research

**DOI:** 10.1186/s12876-017-0641-6

**Published:** 2017-07-20

**Authors:** Yuling Tong, Yulian Wu, Zhenya Song, Yingying Yu, Xinyan Yu

**Affiliations:** 1 International HealthCare Center, The Second Affiliated Hospital of Zhejiang University, School of Medicine, NO.88 Jiefang Road, Hangzhou, Zhejiang Province People’s Republic of China; 2 General Surgery Department, The Second Affiliated Hospital of Zhejiang University, School of Medicine, NO.88 Jiefang Road, Hangzhou, Zhejiang Province People’s Republic of China

**Keywords:** Serum pepsinogen, Atrophic gastritis, *Helicobacter pylori* infection, Health check-up

## Abstract

**Background:**

The aim of this study is to assess the validity of the measurement of pepsinogen as a screening test for chronic atrophic gastritis (AG) in health check-up populations in China.

**Methods:**

Patients from consecutive regular health check-up were enrolled from January 2014 to June 2015. Endoscopy, combined with monitoring the *Helicobacter pylori* (*Hp*) infections, and measuring the serum pepsinogen (PG) were used to determine the diagnostic accuracy of PG for the screening of atrophic gastritis. Histopathology was assessed by the Operative Link on Gastritis Assessment (OLGA) system. Statistical analysis was performed using SPSS statistical software.

**Results:**

The total *Hp* infection rate was 40%. Based on pathology, the 996 participants were divided into three groups: non-atrophic (NAG), mild-moderate atrophic (MAG): stage I and II of the OLGA classification, and severe atrophic (SAG): stage III and IV of the OLGA classification. Compared with NAG and MAG groups, PGR decreased significantly in SAG group (*p* < 0.05). PGI and PGII levels were significantly elevated in *Hp*-positive group, while the PGR was markedly decreased (*p* < 0.01). When MAG and SAG groups were combined and compared with NAG group, the best cutoff value for atrophy diagnosis was PGI ≤50.3 ng/ml; the cutoff value in *Hp*-negative group was absolutely higher than in *Hp*-positive group. When NAG and MAG groups were combined and compared with the SAG group, the best cutoff value for diagnosis of severe atrophy was at PGR ≤4.28. The cutoff values in *Hp*-negative and *Hp*-positive groups were calculated at PGR ≤6.28 and ≤4.28, respectively.

**Conclusions:**

Pepsinogens play an important role in the identification of patients with atrophic gastritis and severe AG. Use of different cutoff values of PG for *Hp*-negative and *Hp*-positive groups may offer greater efficacy in the diagnosis of AG.

**Electronic supplementary material:**

The online version of this article (doi:10.1186/s12876-017-0641-6) contains supplementary material, which is available to authorized users.

## Background

Atrophic gastritis (AG) and intestinal metaplasia (IM) are well-recognized as high-risk conditions for developing gastric cancer (GC), and both have been identified as the precancerous lesions [1–3]. The successive progression from chronic non-atrophic gastritis, by the way of AG and IM, to dysplasia, known as Correa’s cascade [4], is widely known to be a common route of the intestinal type of non-cardia GC. Chances of developing AG, IM, mild-to-moderate dysplasia, and severe dysplasia within 5 years after diagnosis of GC are 0.1%, 0.25%, 0.6%, and 6%, respectively [5]. The Operative Link on Gastritis Assessment (OLGA), based on the histopathology findings of biopsy specimens, gastritis can be effectively ranked into stages with corresponding carcinoma risks [6, 7]. It has reported that a high-risk stage (defined as stage III or IV of the OLGA classification) is strongly correlated with a high risk of GC [8, 9]. Thus, early detection of precancerous lesions of GC in general population and following up with the condition are important considerations for reducing mortality, increasing survival rates, and improving quality of life [10].

The clinical symptoms of AG are not specific. Endoscopy and biopsy are believed to be the reference standards for diagnosis and screening of GC and precancerous lesions of GC. But endoscopy is limited for population-wide screening due to their invasiveness [11–13]. In Japan, the risk for GC and precancerous lesions of GC can be stratified into four groups as follows: group A [*Hp*(−)PG(−)], group B [*Hp*(+)PG(−)], group C [*Hp*(+)PG(+)], and group D [*Hp*(−)PG(+)], according to the *Helicobacter pylori* (*H. pylori* or *Hp*) and the serum pepsinogen (PG) levels, with PGI ≤70 ng/ml and PGR ≤3 classified as PG-positive. It was called ABC method, which was implemented for screening of gastric cancer risk, and has gained great achievement [14–16]. The study found the Hazard ratio for gastric cancer was 9.8 in group B, 19.6 in group C, and 120.4 in group D, respectively. Thus it is recommended that endoscopic examination be performed at least once every 3 years for group B, at least once every 2 years for group C, and annually for group D, and that group A be excluded from the examination [14]. PG are aspartic proteinases, which are mainly secreted by gastric cells, and are classified into two groups: pepsinogen I (PGI) and pepsinogen II (PGII) [17]. PGI is secreted by chief cells and mucous neck cells in the fundic glands, while PGII is secreted by cells in the pyloric and Brunner’s glands [18]. PGI and PGII levels are increased with increasing severity of *Hp*-related chronic gastritis. However, when atrophic changes in the corpus are accompanied by a loss of cells in the corpus, including those secreting PGI, the level of PGI decreases, whereas the level of PGII remains high or stable. Therefore, the PGI/PGII ratio (PGR) decreases in a stepwise manner. More severe atrophy is related to a lower PGR. PG has been proposed as a predictor of various gastric pathologies, including AG and IM [19–21]. However, its efficacy remains controversial, and the cutoff value has varied in different researches. A serum PGI ≤70 ng/ml and PGR ≤3 have been the most widely accepted values for the detection of AG, resulting in a sensitivity of 66.7–84.6% and specificity of 73.5–87.1% [20, 22, 23]. In European countries, the cutoff values in fundic atrophy assessment were estimated at PGI ≤56 ng/ml (sensitivity: 61.9%, specificity: 94.8%); and PGR ≤5 (sensitivity: 75.0%, specificity: 91.0%) [17]. In a Korean study, PGI ≤70 ng/ml showed sufficient sensitivity (72.4%), but a low specificity (20.2%); while the sensitivity and specificity related to a PGR cutoff of ≤3 were 59.2–61.7% and 61.0%, respectively [24]. The participants in most studies were symptomatic patients, and the number of samples was small [25].

PG levels are affected by many factors, such as area, race, age, gender, height, body weight, body surface area, smoking and drinking habits, as well as *H. pylori* infections [26, 27]. Studies have revealed that while the level of PGII increases, PGR decreases in *Hp* infection groups [28, 29]. A recent systematic literature analysis reported that the prevalence of *Hp* infection was 66% in rural Chinese populations and 47% in urban Chinese populations; which exhibits a decreasing trend, but is still higher than other advanced countries [30]. To obtain summary estimates of the diagnostic accuracy of PG for the diagnosis of atrophic gastritis, and to determine whether *Hp*-positive and *Hp*-negative groups should use different cutoff values, we employed endoscopy and the pathology of *Hp* as the “gold standard”, analyzed the serum PG level, and investigated the status of *Hp* infections in health check-up populations at our hospital.

## Method

### Participants

We enrolled subjects, who received consecutive regular health check-up from January 2014 to June 2015 at International Healthcare Center, the Second Affiliated Hospital of Zhejiang University, College of Medicine, Zhejiang Sheng, China. The patients included in this study ranged from 20 to 70 years in age. The exclusion criteria were as follows: patients diagnosed with gastrointestinal tumor and peptic ulcer; patients who underwent gastrectomy or were receiving acid-suppressive drugs; and patients who presented with contraindications of gastroendoscopy. The study was approved and authorized by the hospital’s ethical committee. (Ethical approval number: 2015–082).

### Test methods

All the participants were made to undergo gastroendoscopy and pathology tests, serum PG test, ^13^C–urea breath test or *Hp* serological current infection marker rapid test in one day.

Approximately 5 mL fasting blood was collected from each subject. Blood samples were centrifuged for 10 min at ≥10,000 RCF. Serum PG levels were assayed by chemiluminescent microparticle immunoassay method using Abbott ARCHITECT Pepsinogen I and II Reagent Kit (Abbott Laboratories Inc., Chicago, IL, USA).

Gastrointestinal endoscopy was performed by endoscopists, who had more than ten years of experience, and had no prior knowledge about the serological data of the study subjects. The biopsies were scored semi-quantitatively by two histopathologists, according to the updated Sydney classification system [11], and gastritis was assessed by OLGA system. *H. pylori* infection was detected by rapid urease test.

Each subject was also examined with ^13^C–urea breath test (Shenzhen Zhonghe Headway Bio-Sci & Tech Co. Ltd., P.R.China) or *Hp* serological current infection marker rapid test (MP Biomedicals, Santa Ana, CA, USA). Tests were performed according to the manufacturer’s instructions.

*Hp* infection was determined based on the results of ^13^C–urea breath test or *H. pylori* serological current infection marker rapid test, combined with the pathological screening. Patients showing a positive result for any of the three above mentioned tests were described as *Hp*-positive. If all the tests revealed negative results, the patient was defined as *Hp*-negative.

Based on endoscopic examination and histological appearances, the patients were classified into three categories as follows: non-atrophic gastritis group (NAG group, 852/996), mild-moderate atrophic gastritis group (MAG group, 131/996): stage I and II of the OLGA classification, and severe atrophic gastritis group (SAG group, 13/996) stage III and IV of the OLGA classification.

Data were analyzed after all the tests were completed.

### Statistical analysis

In the normal distribution measurement data, results were expressed as mean ± standard deviation of the mean (mean ± SD). The abnormal distribution measurement data were presented as median ± interquartile range. Statistical analysis was performed using SPSS statistical software (IBM SPSS Statistics 19). The significant differences of age, PGI, PGII level, and PGR among the three groups were assessed by Scheffe test and analysis of variance (ANOVA). *H. pylori* differences were evaluated by Bonferroni correction of Pearson’s chi-square test. ROC curve was used to estimate the cutoff value of PG. A *p*-value <0.05 was considered statistically significant.

## Results

Out of 1074 participants who completed all the serum and gastroendoscopy tests, 75 peptic ulcer patients, one post subtotal gastrectomy patient, one stromal tumor patient, and one esophageal cancer patient were excluded. For the remaining 996 participants (M/F = 1.95:1, with 33.9% females), the mean age was 47.0 ± 8.1 years. The mean age of SAG group was higher than the other two groups. The total prevalence rate of *Hp* infection was 40.0% (398/996). *Hp* infection showed significant differences between MAG group and NAG group (Table 1).

**Table 1:** The mean age and differences in *H. pylori* infection among the NAG group, MAG group, and SAG group

	NAG group	MAG group	SAG group
N,%	852, 85.5%	131, 13.2%	13, 1.3%
Gender			
Male	564	89	5
Female	288	42	8
Age (years)	46.4 ± 8.0*	49.7 ± 7.6*	56.9 ± 9.4
P	0.001	0.005	
*H. pylori* infection			
Positive	321	71	6
Negative	531	60	7

*p < 0.01, compared with SAG group

### Serum PG differences among the groups

Within the NAG group, serum PGI and PGII levels were significantly elevated in the *Hp*-positive group, while the PGR was apparently decreased (*p* < 0.01). The same pattern of differences was found in the MAG group and SAG group. Compared with NAG and MAG groups, PGR was significantly decreased in SAG group, under the same *Hp* infection conditions (*p* < 0.05; Table 2).

**Table 2:** Serum PG levels and differences in PGI/II ratio among the NAG group, MAG group, and SAG group, layered by *H. Pylori* infection

	NAG group		MAG group		SAG group	
	*Hp-*(*n* = 531)	*Hp+*(*n* = 321)	*Hp-*(*n* = 60)	*Hp+*(*n* = 71)	*Hp-*(*n* = 7)	*Hp+*(*n* = 6)
PGI(ng/ml)	46.0 ± 19.8	60.6 ± 33.1^*^	50.7 ± 24.4	62.7 ± 30.0^*^	41.6 ± 10.3	78.4 ± 53.4^*^
PGII(ng/ml)	6.3 ± 3.3	13.2 ± 9.2^*^	6.8 ± 3.6	14.2 ± 11.3^*^	10.5 ± 4.4^**^	22.1 ± 14.8^*^
PGR	7.3 ± 2.3	4.9 ± 2.4^*^	7.9 ± 2.1	4.7 ± 2.7^*^	4.5 ± 2.2^#**^	3.6 ± 0.6^#***^

*XHp-*: *Hp* negative group; *Hp+*: *Hp* positive group; *p < 0.01, compared with *Hp*-negative groups; #*p* < 0.05, compared with NAG group with same *Hp*infection condition; **p < 0.05, compared with MAG group with the same *Hp* infection condition

### Diagnostic value of PG for atrophy

The MAG and SAG groups were combined into one group, namely the atrophy group, and were further compared with the NAG group. Subsequently, the optimal cutoff values for PGI levels and PGR were investigated for the diagnosis of atrophy. The best cutoff value for atrophy assessment was estimated at PGI ≤50.3 ng/ml (sensitivity: 63.9%, specificity: 49.1%; positive predictive value: 10.9%, negative predictive value: 82.5%).

When the *H. pylori* infections were taken into consideration, the best cutoff values among the *Hp*-negative group for atrophy diagnosis were calculated at PGI ≤41.2 ng/ml (sensitivity: 77.6%, specificity: 35.4%) and PGR ≤9.08 (sensitivity: 29.9%, specificity: 82.9%). For *Hp*-positive group, the best cutoff values were PGI ≤43.4 ng/ml (sensitivity: 89.6%, specificity: 18.1%) and PGR ≤4.29 (sensitivity: 49.4%, specificity: 65.1%) (Table 3). However, these results had no statistical significance (*p* > 0.05).

**Table 3:** Predicting atrophy based on serum PGI levels and PGR

	Cut-off value	Sensitivity (95%CI),%	Specificity (95%CI),%	PPV,%	NPV,%	AUC (95%CI)	Accuracy,%	p
Mild-moderate atrophy	PGI ≤ 50.3 ng/ml	63.9 (55.5–71.7)	49.1 (45.7–52.5)	10.9	82.5	0.575 (0.544–0.606)	49.0	0.0022
	PGR ≤ 5.15	41.7 (33.5–50.2)	74.5 (71.5–77.4)	21.7	88.3	0.556 (0.524–0.587)	69.8	0.0516
	*Hp* negative							
	PGI ≤ 41.2 ng/ml	77.6 (65.8–86.9)	35.4 (31.3–39.6)	7.4	86.8	0.568 (0.527–0.608)	59.9	0.0584
	PGR ≤ 9.08	29.9 (19.3–42.3)	82.9 (79.4–86.0)	9.7	82.0	0.513 (0.472–0.553)	23.1	0.7570
	*Hp* positive							
	PGI ≤ 43.4 ng/ml	89.6 (80.6–95.4)	18.1 (14.0–22.7)	12.1	79.2	0.521 (0.471–0.571)	68.1	0.5545
	PGR ≤ 4.29	49.4 (37.8–61.0)	65.1 (59.6–70.3)	25.3	84.3	0.544 (0.493–0.593)	62.1	0.2629
Severe atrophy	PGI ≤ 53.6 ng/ml	76.9 (46.2–95.0)	47.0 (43.8–50.2)	1.9	97.3	0.575 (0.544–0.606)	47.4	0.3774
	PGR ≤ 4.28	76.9 (46.2–95.0)	83.4 (80.9–85.7)	5.8	99.6	0.818 (0.793–0.842)	83.3	<0.0001
	*Hp* negative							
	PGI ≤ 53.6 ng/ml	100.0 (59.0–100.0)	33.7% (29.9–37.6)	1.8	100.0	0.635 (0.595–0.673)	34.4	0.1696
	PGR ≤ 6.28	85.7 (42.1–99.6)	73.9 (70.2–77.4)	37.5	99.8	0.833 (0.801–0.862)	75.0	0.0001
	*Hp* positive							
	PGI ≤ 102.9 ng/ml	66.7 (22.3–95.7)	9.7 (7.0–13.1)	1.0	94.9	0.522 (0.472–0.572)	10.5	0.8856
	PGR ≤ 4.28	100.0 (54.1–100.0)	64.3 (59.3–69.0)	4.1	100.0	0.789 (0.746–0.828)	63.3	<0.0001

*CI* confident interval, *AUC* area under curve, *PPV* positive predictive value, *NPV* negative predictive value

### Diagnostic value of PG for severe atrophy

Next, the NAG group and MAG group were combined into one group, and were compared with the SAG group. The best cutoff value for severe atrophy diagnosis was calculated at PGR ≤4.28 (sensitivity: 76.9%, specificity: 83.4%, positive predictive value: 5.8%, negative predictive value: 99.6%).

On considering the *H. pylori* infections, the best cutoff values among *Hp*-negative group for the diagnosis of severe atrophy was determined at PGR ≤6.28 (sensitivity: 85.7%, specificity: 73.9%, positive predictive value: 37.5%, negative predictive value: 99.8%); while for *Hp*-positive group, the best cutoff value was PGR ≤4.28 (sensitivity: 100%, specificity: 64.3%, positive predictive value: 4.1%, negative predictive value: 100%) (Table 3) (Additional file 1).

## Discussion

Atrophic gastritis (AG) and its causative etiological agent, *Helicobacter pylori* (*Hp*), are well-established precursor lesions of non-gardia GC [1–3, 31–33]. In the present study, 14.5% of the total cases were diagnosed with AG; more specifically, 1.3% of the cases presented with severe AG (stage III and IV of the OLGA classification) in the asymptomatic health check-up populations. It has been stipulated that nearly 0.1% AG develops into GC within 5 years of diagnosis [5], and a high-risk stage (defined as stage III or IV of the OLGA classification) is strongly correlated with a high risk of GC [8, 9]. Detecting chronic AG in general population and following up with patients has a great significance in terms of early diagnosis.

PGI is produced in the fundic glands and decreases proportionally with progression of fundic atrophy. PGII is synthesized in most parts of the gastric mucosa and part of the duodenum, and shows no consistent pattern with fundic or antral atrophy, although decrease in PGR has been associated with detection of fundic atrophy [19, 34, 35]. A serum PGI level of ≤70 ng/ml and PGR ≤3 was found most appropriate, and showed promising results in the detection of AG [20, 22, 23]. Nevertheless, many other studies employed different SPG cutoff values and different SPG analytical technologies, and thereby exhibited different sensitivities and specificities [36–38]. In our study, the mean PGR was found to be above 3, even in the *Hp*-positive SAG group (3.6 ± 0.6). For atrophy diagnosis, PGI ≤ 50.3 ng/ml showed sufficient sensitivity (63.9%) and negative predictive value (82.5%), but a low specificity (49.1%) and positive predictive value (10.9%). While for severe atrophy diagnosis, PGR ≤ 4.28 showed high sensitivity (76.9%) and specificity (83.4%). Pepsinogens (PG) could, therefore, be used as a potential biomarker for the diagnosis of AG, particularly severe AG, in large-scale screening. Considering the different cutoff values with other studies, participants (asymptomatic health check-up population vs. patients with symptoms and past gastritis histories) might be one of the reasons. And the differences of *Hp* infection rate might be another important reason.

In the present study, *H. pylori* infections were tested by ^13^C–urea breath test or *Hp* serological current infection marker rapid test, and not by the serum anti-*Hp* IgG antibody. Therefore, the results showed more accuracy. The total *Hp* infection rate in our study was lower (40.0%) than the population-based screening analysis in St. Petersburg (76.7%) [32] and hospital-based screening study in Astana (76.5%) [39]; but our data were in sharp contrast to those recently reported in two Nordic countries (Finland and Sweden), where the overall prevalence of *Hp* infection was only 19% [40] and 28.7% [41], respectively. *Hp* infection has been shown to significantly aid in the progression of gastric mucosal inflammation and development of IM and AG [42–45]. In our study, *Hp* prevalence was predominantly higher in the MAG group (54.2%). For the asymptomatic participants in the MAG group, *Hp* infection might turn out to be the main reason resulting in the atrophy.

Furthermore, PGII was found to increase two-fold in the present study; this change was much greater than observed for PGI in *Hp*-positive participants, and caused an apparent decrease in PGR. These findings were similar to the results published by other researchers [29, 36, 46]. PGI, secreted by chief cells and mucous neck cells in the fundic glands, is inversely correlated with atrophy and IM in the corpus; whereas PGII, secreted by cells in the pyloric and Brunner’s glands, acts as a marker of gastric inflammation for the whole stomach (that is, the antrum and corpus) [18]. *Hp* infection promoted the secretion of gastrin, which in turn caused the elevated secretion of PGI and PGII. However, at a later stage, the loss of cells in the corpus, including the PGI-secreting cells, led to a decrease in the level of PGI, while the level of PGII remained high or stable.

Additionally, we calculated the sensitivity and specificity of PG based on the status of *Hp* infections. The cutoff value in *Hp*-negative group (PGR ≤9.08) was absolutely higher than in *Hp*-positive group (PGR ≤4.29) for atrophy diagnosis; but the difference was not statistically significant (*p* > 0.05). Nevertheless, for the diagnosis of severe atrophy, the best cutoff value among *Hp*-negative and *Hp*-positive groups was estimated at PGR ≤6.28 and ≤4.28, respectively. The AUC value of these two cutoff points approaching 0.850 is an indication of an excellent sensitivity/specificity balance of this biomarker as a predictor of severe atrophy. We, therefore, proposed that employing different cutoff values of PG for *Hp*-negative and *Hp*-positive groups might be more useful in the diagnosis of AG and severe AG. And for screening of the risk for GC and precancerous lesions of GC by ABC method, it might need to use different cutoff value of PG for PG-positive definition, in *Hp*-negative (group A and group D) and *Hp*-positive groups (group B and group C), then to recommend the frequency of endoscopy.

The current study had certain limitations. Since the participants were selected from an asymptomatic health check-up and there was a time limit to the study, the sample size of SAG group was too small. In addition, the PG levels are affected by many other factors, such as area, race, age, gender, height, body weight, body surface area, as well as smoking and drinking habits [26, 27], which were not taken into consideration in this study. Thus, further investigation should comprise larger number of samples so as to include more severe atrophy patients; and the baseline of body weight, smoking habits, drinking habits, etc., should be balanced. Our next study will extend the populations, and we have already begun the early gastric cancer and precancerous lesions screening in multi-centers throughout the country, and take the follow up for those objects by ABC method, using the cutoff value of PG established by this study, to validate these conclusions.

## Conclusions

In conclusion, serum pepsinogens is a cost effective screening method in the identification of patients with AG, especially severe AG, which are precancerous lesions of GC. For ABC method, The PG-positive definition might use different cutoff values of PG in *Hp*-negative (group A and group D) and *Hp*-positive (group B and group C) groups, to recommend the frequency of endoscopy. Large-scale and well-designed prospective studies need to be undertaken for further discuss and validate the cutoff values for *Hp*-negative and *Hp*-positive groups, in order to accurately estimate the low-risk group and high-risk group for the screening of gastric cancer.

## Additional file

Additional file 1: Datasets in more detail. The first column refers to the group: the number “0” represents the “non atrophic group (NAG)”, the number “1” represents the “mild-moderate atrophic group (MAG)”, and the number “4” represents the “severe atrophic group (SAG)”. The second column refers the age of patients. The third column refers the condition of Helicobacter pylori infection. The number “1” represents “*Hp* positive”, and the number “0” represents “*Hp* negative”. The last three columns refers the PGI levels, PGII levels and the PGI/PGII ratio. (XLS 3615 kb)

## Abbreviations

AG: Atrophic gastritis
GC: Gastric cancer
*Hp*: *Helicobacter pylori*
IM: Intestinal metaplasia
MAG: Mild-moderate atrophic
NAG: Non-atrophic
PG: Pepsinogen
PGI: Pepsinogen I
PGII: Pepsinogen II
PGR: PGI/PGII ratio
SAG: Severe atrophic
